# *De novo* assembly of the *Platycladus orientalis* (L.) Franco transcriptome provides insight into the development and pollination mechanism of female cone based on RNA-Seq data

**DOI:** 10.1038/s41598-019-46696-6

**Published:** 2019-07-15

**Authors:** Wei Zhou, Qi Chen, Xiao-Bing Wang, Tyler O. Hughes, Jian-Jun Liu, Xin Zhang

**Affiliations:** 1 College of Landscape Architecture and Arts, Northwest A&F University, Yangling, Shaanxi P.R. China; 2 School of Life Science and Technology, Xinxiang University, Xinxiang, Henan P.R. China; 3 Department of Biology, The Pennsylvania State University, University Park, PA 16802 USA; 4 Key Laboratory of Silviculture on the Loess Plateau State Forestry Administration, College of Forestry, Northwest A&F University, Yangling, P.R. China

**Keywords:** Plant physiology, Flowering, Pollination

## Abstract

For seed-bearing plants, the basis of seed and fruit formation is pollination. The normal progression of pollination is through advances in continuous signal exchange and material transfer, which occur mainly in female reproductive organs; thus, the molecular mechanism of development in female reproductive organs is vital for understanding the principle of pollination. However, molecular biology studies on the development of female cones related to pollination are rare and unclear in gymnosperms, especially in Cupressaceae. In this study, *Platycladus orientalis*, a monotypic genus within Cupressaceae, was chosen to examine female cone transcriptomes at pre-pollination and pollination stages by Illumina paired-end sequencing technology to *de novo* sequence six libraries with 3 biological replicates. These libraries were used to construct a *P*. *orientalis* transcriptome database containing 71,669 unigenes (4,963 upregulated unigenes and 11,747 downregulated unigenes at the pollination stage) for subsequent analysis. Based on the annotations and expression levels, the functions of differentially expressed unigenes and enriched pathways between the developmental processes of female cones were analysed to detail the preliminary development and pollination mechanism of the female cone. Targeted investigations were specifically performed to determine the elementary mechanism of secretion and functioning of the pollination drop, a vital ovule secretion at the pollination stage. Ultimately, the expression of 15 unigenes selected between two stages were further assessed and confirmed using qRT-PCR, which demonstrated reliable data and significant differences in the expression profiles of key genes. As one of the largest available transcriptomic resources of this species, the database is constructed to prospectively adapt to the physiological and genomic data of woody plants. This work provided the first transcriptome profile of *P*. *orientalis* female cones at different developmental stages, and will promote the illumination of the pollination mechanism of *P*. *orientalis*, and will serve as the basis for in-depth genomic study in the Cupressaceae family. This initiative will arouse the interest and attention of scholars and pave the way for future studies.

## Introduction

Pollination is a key procedure in the amphimixis of seed species. The development of the female reproductive organ is a critical and complicated process in plant growth and an essential premise in pollination, concerning interactions among multiple endogenic and exogenic factors^1^. There are many practical experiences with respect to reproductive control, which is an important part of the conifer genetic improvement and seed productive process. However, relevant information in molecular biology on the development and regulation of female cones of conifers is still scarce. In addition, the structures of reproductive organs of gymnosperms are substantially different from that of angiosperms, such as mostly monoecism and lacking perianth and carpel, which leads to that many important achievements in reproductive physiology of angiosperms are difficult to support the related researches on gymnosperms^2^. Improving knowledge about the molecular aspects of development in female cones in gymnosperms is an essential tool to determine how female cones develop and to evaluate the biological pathways and vital genes that participate in the development and pollination processes.

Several metabolic and signalling pathways and a great number of genes have been determined in plant reproductive process, and some of these genes distinctly control, mediate and facilitate plant reproduction^1,3–6^. However, similar studies of gymnosperms are still limited, and the molecular mechanisms controlling reproduction remain largely unknown.

It is worth mentioning the pollination drop, a special liquid secretion that most gymnosperms produce during the reproductive process, as a critical and indispensable secretion for successful pollination. The pollen attaches to the sticky drop and is pulled into the micropyle of the ovule with retraction of pollination drop. This pollination mechanism highly improves the pollination efficiency of gymnosperms^7^. Due to the absence of a connection to the vascular system^8^, the pollination drop is not apparently related to osmotic potential of the xylem, and it is usually believed to be secreted apoplastically from secretion cells at the nucellus tip of the ovule^9,10^ and affected by the outside environment^9,11,12^. Previous researchers have mainly focused on investigations of the creation and retraction of the pollination drop, not involving the physiological pathway and process^9,11,13–15^. The mechanism governing pollination drop secretion remains unexplained. Knowledge concerning the mechanisms of the movement and function of the pollination drop is still in its infancy^16–19^.

*Platycladus orientalis* (L.) Franco is a monotypic species of *Platycladus* in Cupressaceae and a widespread and ecologically important native tree in China. This species is widely used for silviculture and ornamental landscape due to its beautiful shape, evergreen leaves and air purification ability^20^, and it is also extensively applied in wood industries and traditional Chinese medicine^21^. However, genomic resources for the study of *P*. *orientalis*, a non-model plant species, are relatively scarce^20,22^, and the molecular mechanism underlying the reproductive processes of *P*. *orientalis* female cones remain unknown.

The transcriptomic techniques used allowed for the assessment of the complexity of the female cone transcriptomes and the prediction of putative functions for the differentially expressed genes (DEGs) in developmental process of the female cone. Transcriptomic data from different stages of female cone development will advance the understanding of gymnosperm reproductive development. Moreover, transcriptomic studies in gymnosperms are needed to better grasp their pollination mechanism. Understanding the expression profile of *P*. *orientalis* at the transcriptome level and its changes before and during pollination will not only provide important reference information for studying the function of the *P*. *orientalis* cone during pollination but also contribute to improving the pollination efficiency of *P*. *orientalis* through the use of genetic engineering technology to improve its practical significance. This is the first transcriptome assembled from the female cone of *Platycladus* at different developmental stages, and the first transcriptome focused on the development and pollination mechanism of female cones in gymnosperms.

## Methods

### Sampling and collection

The trees of *P*. *orientalis* were sampled in the park of Yellow Emperor Mausoleum at Huangling county, Yan’an city, Shaanxi province, China (35°35.102′ N, 109°16.218′ E). Six proximal individuals with the same tree height, crown width, health status, age (30 years) and environmental condition were selected from thousands of *P*. *orientalis* and divided into 2 groups (including three independent biological replicates each) for female cone collection at the two stages (Table 1). The eligible female cones were cut off and immediately placed into the storage tube filled with RNAlater (RNA Stabilization Solution, Solarbio, Beijing, China). The volume of female cones was maintained under one-tenth of RNAlater. When the collection was performed at the pre-pollination stage, the target female cones of the pollination stage were selected and bagged to avoid pollen pollution or contamination. After the collection, the tubes were stored overnight (4 °C) in compliance with the RNAlater manufacturer’s instructions. Then, the tubes were transferred into liquid nitrogen for freezing until they were returned to the lab and were stored at −80 °C for RNA-Seq and qRT-PCR analysis.

**Table 1:** Presentation of the sample collection.

Group	Replicas	Stage	Collection time	Collection date	Direction
1	3 trees	Pre-pollination	7 a.m. to 10 a.m.	Feb. 20, 2016	South
2	3 trees	Pollination	7 a.m. to 10 a.m.	Mar. 28, 2016	South

### Transcriptome sequencing

Total RNA of *P*. *orientalis* for each sample listed in Table 1 was extracted using the TRIzol Kit (Promega, USA) following the procedure manuals. Then, the total RNA was treated with RNase-free DNase I (Takara Bio, Japan) for 30 min at 37 °C to remove residual DNA. The RNA quality was verified using a 2100 Bio-analyser (Agilent Technologies, Santa Clara, CA) and was checked by RNase free agarose gel electrophoresis. Then, poly(A) mRNA was isolated and enriched using oligo-dT magnetic beads (Qiagen, Germany). All mRNA was broken into short fragments by addition of fragmentation buffer. First-strand cDNA was generated using random hexamer-primed reverse transcription following the synthesis of the second-strand cDNA using RNase H and DNA polymerase I. The cDNA fragments were purified using a QiaQuick PCR extraction kit. These purified fragments were then washed with EB buffer for end reparation poly(A) addition and ligated to sequencing adapters. Following agarose gel electrophoresis and extraction of cDNA from gels, the size-selected cDNA fragments were purified and enriched by PCR amplification to construct the final cDNA libraries. To identify DEGs of two stages, digital gene expression (DGE) profiling analyses were performed with the 6 libraries which were defined as follows: Befl-1, Befl-2 and Befl-3 as replicates for the pre-pollination stage, and Fl-1, Fl-2 and Fl-3 for the pollination stage. The assembled reference library was built by blending evenly of RNA from the 6 samples together.

The cDNA libraries were sequenced separately using paired-end technology on Illumina HiSeq™ 4000 system of Gene Denovo Co. (Guangzhou, China).

### Reads filtering and alignment and normalization of gene expression levels

A Perl script was created to choose clean reads by eliminating low-quality reads (containing over half of nucleotides with low quality (Q-value ≤ 10) in a read), reads with exceeding 5% unknown nucleotides and reads including adaptors from raw sequences. Next, the clean reads were aligned to the sequences of rRNA to detect the remaining rRNA reads. The high-quality clean reads with rRNA removed were aligned to reference data using SOAPaligner/soap2, a software of read alignment, and the mapping ratio was calculated. No more than 2 mismatched bases were permitted. Then, the *de novo* assembly was performed with a sequence assembling tool – Trinity^23^. Assembled by reads overlap, these fragments without N base are called unigenes.

Coverage of reads in a unigene was employed to represent the abundance of said unigene. The expression levels of all unigenes were acquired in this way. Reads, exclusively aligned to a unigene, were employed to measure the unigene expression levels which were calculated by a normalizing statistic called the number of exclusively mapped fragments per kilobase of exon region per million mappable reads (RPKM)^24^ which is defined as follows:

$${\boldsymbol{RPKM}}=\frac{{10}^{6}C}{NL/{10}^{3}}$$

where C is the number of reads which are exclusively mapped to the specific unigene, N is the number of reads which are exclusively mapped to the total unigenes, and L is the exon size of the specific unigene. The means of RPKM excludes the effect of unigene lengths and the differences in throughput of sequencing. Thus, the RPKM value was employed to compare the discrepancies of unigene expression levels of different libraries. Statistics and visualizations of the expression levels were performed with the R package.

### Repeatable test of replicas

Correlation analysis of parallel libraries provides an assessment of the operational stability and experimental dependability. The Principal component analysis (PCA) and correlation coefficient of expression profiling data among samples were measured to assess the repetitiveness among replicates. To ascertain the segregation of expression patterning across replicates and reveal the relationship of the samples, we performed PCA at all unigene levels. The PCA was performed with “fast.prcomp” of R on centred but unscaled data.

Unigenes with analogous expression profiles always indicate a functional correlation. Clustering analysis of unigene expression profiles was conducted with “heatmap.2” in the gplots package of R and the results were showed as a heatmap.

### Annotation of unigenes

The assembled unigenes were annotated using the BLASTx tool with E-value < 1e^−5^ against the NCBI non-redundant protein (nr) database, Swiss-Prot protein database, Kyoto Encyclopaedia of Genes and Genomes (KEGG) database, and eukaryotic Cluster of Orthologous Groups of proteins (KOG) database. When the results of different databases were inconsistent, the final outcome was confirmed in priority of nr, Swiss-Prot, KEGG and KOG. The best outcomes were then selected to determine the protein functional annotations and sequence direction. When a unigene cannot be annotated to any of the four protein databases, the ESTscan program was operated to confirm the protein-coding sequence and direction.

Gene Ontology (GO) annotations of the unigenes were analysed using Blast2GO software based on the nr database. WEGO software was used to determine the functional categorisation. KEGG pathways were annotated using Blastall tool against the KEGG database.

### Identification of DEGs and functional enrichment analyses of GO and KEGG

With the basis of normalization of unigene expression levels, differential expression analysis was performed with edgeR. The *p*-value threshold was evaluated by false discovery rate (FDR) in multiple tests. FDR < 0.05 and |log_2_Ratio| > 1 were employed to determine the significance of the DEGs between Befl and Fl.

The enrichment analyses of GO and KEGG were then performed with DEGs. First, all the DEGs were aligned to GO terms based on the GO database. Second, unigene numbers were measured for every term. Finally, compared to the genome background, significantly enriched GO terms were determined by the hypergeometric test. The formula used to calculate the *p*-value was as follows:

$${\boldsymbol{P}}=1-\mathop{\sum }\limits_{{\boldsymbol{i}}=0}^{{\boldsymbol{m}}-1}\frac{(\begin{array}{c}M\\ i\end{array})(\begin{array}{c}N-M\\ n-i\end{array})}{(\begin{array}{c}N\\ n\end{array})}$$

where N is the number of the total unigenes which are annotated to a GO term, n is the number of DEGs in N, M is the number of the total unigenes which are annotated to certain GO terms, and m is the number of DEGs in M. The calculated *p*-value was subjected to Bonferroni correction, with a corrected *p*-value threshold of 0.05. GO terms meeting the setting were selected as significantly enriched GO terms. The calculation of KEGG pathway enrichment analysis was the same as that for GO. GO terms and KEGG pathways with a *Q*-value threshold of 0.05 were significantly enriched according to the means analogous to that explained by Zhang *et al*.^25^.

### Simple sequence repeat (SSR) locus detection and primer design

MIcroSAtellite (MISA) Perl script was used for microsatellite digging in the whole transcriptome of *P*. *orientalis*. The parameters were evaluated to detect the ideal minimum thresholds of the nucleotides specified by the repeat units during the SSR analysis of 6, 5, 4, 4 and 4 for di-, tri-, tetra-, penta-, and hexa-nucleotide motifs, respectively. If the distance between two SSRs was shorter than 100 bp, then they were considered as one SSR. Based on the MISA results, Primer3 software was employed to design primer pairs in the flanking areas of SSRs with default parameters.

### qRT-PCR validation

To validate the reliability of the RNA-Seq experiments, a total of 15 candidate DEGs (average RPKM greater than 50 and read counts of sequencing depth greater than 20) that were highly related to the development and pollination of the female cone were selected for qRT-PCR. Total RNA was isolated from the female cone tissues in three biological replicates. cDNA synthesis was performed using Hiscript qRT superMix (Vazyme, Biotech) as described by the manufacturer using an oligo(dT) primer and 200 ng of total RNA. The samples were then treated with RNaseH (Promega, USA) and stored at −20 °C. All primers were designed using Primer 5.0 software (Premier, Canada) and are listed in Supplementary Table S10. The quantitative reaction was performed on a LightCycler 96 real-time PCR system (Roche, Germany) using SYBR Real-Time PCR Premix (Bioteke, China). PCR amplification was performed under the following conditions: 2 min at 95 °C, followed by 45 cycles of 95 °C for 10 s, 55 °C for 20 s and then 72 °C for 20 s. Three independent biological replicates were performed for each stage, with three technical replicates of each sample. The relative expression levels of the selected genes were calculated using the 2^−ΔΔCT^ method^26^. Relative mRNA levels were quantified with respect to the reference gene ‘*UBC* (Ubiquitin-conjugating enzyme E2)’ of *P*. *orientalis* selected according to Chang’s method^20^. To compare the RNA-Seq and qRT-PCR data, the fold changes (FC) in the gene expression levels of the pollination samples were calculated relative to the pre-pollination samples used for the RNA-Seq and qRT-PCR analyses.

## Results

### Sequence and assembly

To acquire the transcriptome of female cones at different growth stages, RNA samples from female cones of pre-pollination and pollination stages were used for RNA-Seq analysis. After construction and filtration, high-quality clean reads (45.5–53.3 million 150-bp paired-end reads) in each of the libraries passing the saturation analysis and randomness statistics were generated with a Q20 percentage exceeding 97% (Table 2).

**Table 2:** Statistics of RNA-seq of the DGE libraries after filtration.

Sample name Clean data	Number of high-quality reads	Bases (bp)	Containing N (%)	Q20 (%)	Q30 (%)	GC (%)
Befl-1	53,264,694	7,962,934,317	0.00927	97.35	93.64	44.10
Befl-2	48,877,588	7,306,118,492	0.00973	97.03	93.01	43.57
Befl-3	45,471,556	6,798,195,975	0.00948	97.41	93.75	43.19
Fl-1	49,466,976	7,395,889,482	0.00921	97.48	93.88	44.37
Fl-2	52,503,690	7,849,012,114	0.00908	97.25	93.40	44.45
Fl-3	49,742,792	7,435,931,194	0.00931	97.37	93.68	45.07

6 DGE libraries were constructed from pre-pollination (Befl) and pollination (Fl) stages of *P*. *orientalis* female cones. Each stage had 3 independent replicates. Q20 (%) and Q30 (%) demonstrates the percentage of sequences with sequencing error rate lower than 1% and 0.1%, respectively. Containing N (%) demonstrates the percentage of unidentified bases. GC (%) represents the percentage of G and C bases.

There were 128,747 transcripts which were created with a mean size of 1,199.20 bp and a N50 length of 1,971 bp upon Trinity package *de novo* assembly of the qualified clean reads. The longest transcript was selected to represent the unigene when it can be assembled as different length transcripts. As a result, 71,669 unigenes with good gene coverage were acquired. The average length was 908.36 bp (Table 3), and the length distributions of the unigenes are illustrated in Supplementary Fig. S1. Reliable repeatability was demonstrated by PCA, a correlative heat map, Pearson correlation and expression profile clustering analysis of each DGE library.

**Table 3:** Summary of the assembly.

	Unigenes	Transcripts
Number of sequences	71,669	128,747
Number of assembled bases (bp)	65,101,159	154,393,277
Percent GC	40.09	40.36
Largest transcript (bp)	16,379	16,379
Smallest transcript (bp)	201	201
Average length (bp)	908.36	1,199.20
N50 (bp)	1,621	1,971

The N50 represents the median of the contig-size that is the last contig length when the additive length reaches half of the total contig-size after sorting the contigs by size.

### Characterization of SSRs and primer design

**Figure 1:**
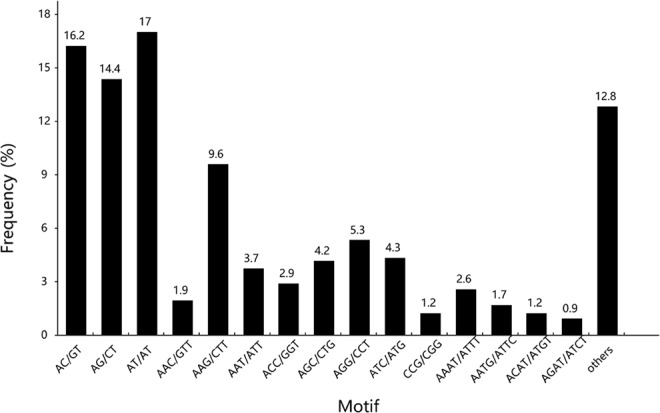
Statistics of the SSRs identified from the *P*. *orientalis* transcriptome. The abscissa represents the motif types of SSRs, and the ordinate represents the frequency of each type.

The assembled transcript library herein indicates an abundant resource for possible SSR markers. A total of 2,840 SSRs of 154 motif types were determined in 2,477 unigenes (Supplementary Table S1). Of these, 284 (11.47%) unigenes included two SSRs or more. The most abundant type of SSRs was di-nucleotides, accounting for 47.75% (1,356), followed by tri-nucleotide (969, 34.12%) repeat motifs. AT/AT (483), AC/GT (461) and AG/CT (408) occupied 35.62%, 34.00% and 30.09% of the di-nucleotide repeats, and AAG/CTT (272) and AGG/CCT (151) accounted for 28.07% and 15.58% of the tri-nucleotides, respectively. Small numbers of tetra-nucleotides (293, 10.32%), penta-nucleotides (63, 2.22%) and hexa-nucleotides (159, 5.60%) were also identified (Fig. 1). The frequencies of SSRs with 6 tandem repeats (746, 26.27%) were most common, followed by 5 (687, 24.19%), 4 (414, 14.58%), 7 (341, 12.01%), and 8 tandem repeats (212, 7.46%). Using Primer3, 3 primer pairs were designed for each designable SSR locus in the flanking regions of the SSR. Supplementary Table S2 shows the unigenes with SSRs and the properties of designed primers.

### Annotation of the reference transcriptome

**Figure 2:**
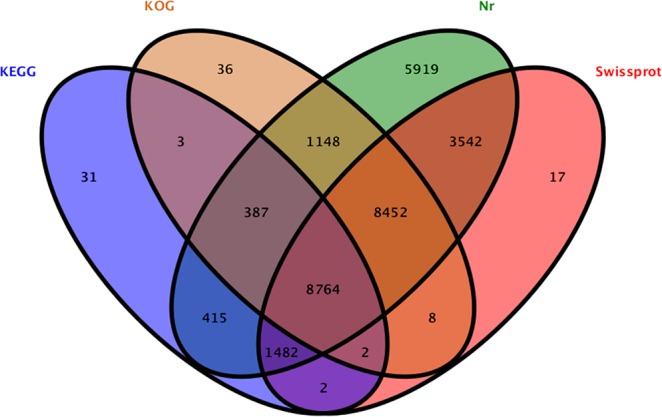
Venn diagram statistics for the number of unigene sequences subjected to BLASTx against the four public databases with an E-value threshold of 10^−5^. Numbers in the areas with different colours represent the number of unigenes annotated by one or several databases.

These unigenes were annotated against the NCBI nr and Swiss-Prot protein databases and searched by BLASTx with an E-value threshold of 10^−5^. As a result, 30,109 unigenes (42.0%) were annotated using NCBI nr database. 22,269 unigenes (31.1%) had significant identities with proteins in Swiss-Prot database. Moreover, 18,800 and 11,086 unigenes were annotated via alignment according to the KOG and KEGG databases, respectively. To sum up, 30,208 unigenes (42.15%) were successfully identified by at least one of the four public databases, with 8,764 unigenes (12.23%) annotated on a homologue in four databases and the remaining 41,461 unigenes (57.85%) potentially undefined or specific to *P*. *orientalis* (Fig. 2).

**Figure 3:**
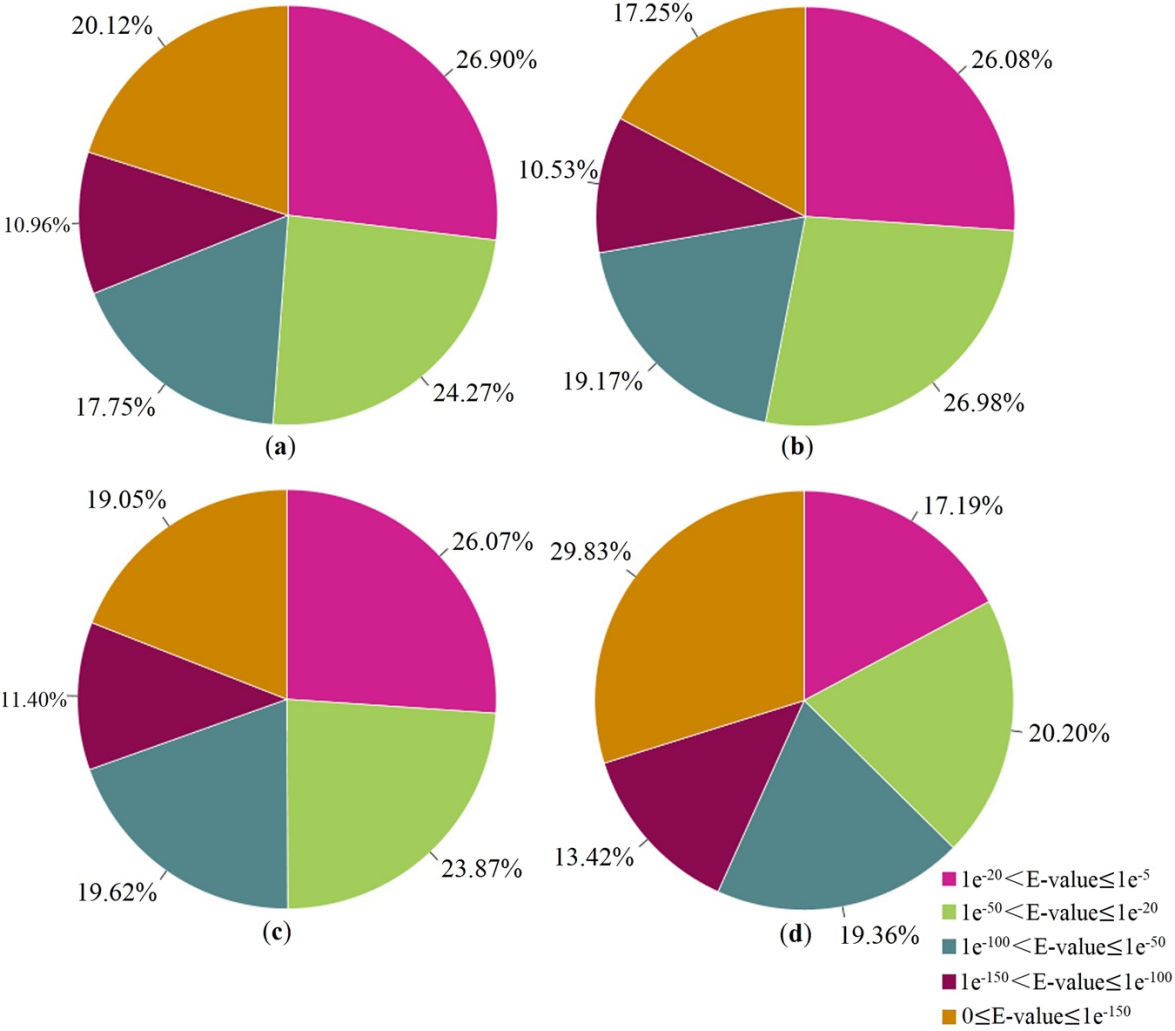
E-value classifications of the BLASTx hits against the four databases. (**a**–**d**) Show the distributions of the E-value in NCBI nr, Swiss-Prot, KOG, and KEGG, respectively.

Based on the four databases, 17.19–26.90% of the unigenes demonstrated homology (1e^−20^ < E-value < 1e^−5^), 39.56–46.15% of which demonstrated strong homology (1e^−100^ < E-value < 1e^−20^) and the rest of 27.78–43.25% demonstrated extremely strong homology (E-value < 1e^−100^) to the accessible plant sequence resources (Fig. 3).

### Functional prediction and categorisation of transcriptomic data by GO and KOG

**Figure 4:**
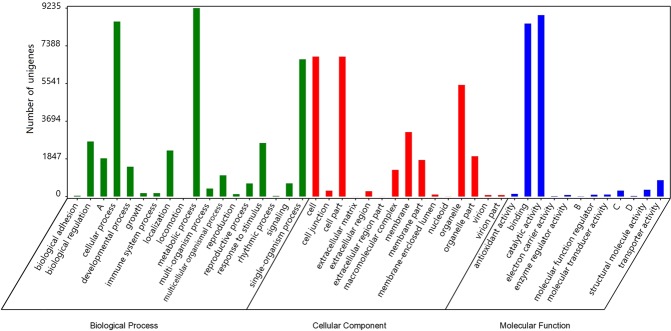
Categorisation of the identified unigenes depending on GO functional classification. They are classified into 45 GO classes of three ontologies: biological process, cellular component, and molecular function. The abscissa represents the ontologies and classes, and the ordinate represents the unigene number for a specific category of GO classes. A, B, C and D on the abscissa represent cellular component organization or biogenesis, guanyl-nucleotide exchange factor activity, nucleic acid binding transcription factor activity, and protein binding transcription factor activity, respectively. The statistics for each bar were independent.

The GO and KOG databases were used to predict and categorised the function of all the unigenes. In total, 71,669 unigenes were aligned to GO terms. The term is the basic unit of GO. Each term corresponds to an attribute. As a result, 17,403 unigenes were categorised into 2,100 terms in the GO database. (Supplementary Table S3). They were classified into 45 classes of three GO ontologies, including cellular component (CC), molecular function (MF), and biological process (BP), based on the sequence homology (Supplementary Table S3). The distribution of GO classes for BP, MF and CC is presented in Fig. 4, and the height of columns represents the number of unigenes. For the BP ontology, the classes “metabolic process” (GO: 0008152), “cellular process” (GO: 0009987) and “single-organism process” (GO: 0044699) showed a significantly higher representation than the other classes. For the MF ontology, “catalytic activity” (GO: 0003824) was the most highly represented class, followed closely by “binding” (GO: 0005488). For the CC ontology, the major classes were “cell” (GO: 0005623), “cell part” (GO: 0044464) and “organelle” (GO: 0043226) (Fig. 4 and Supplementary Table S3). These annotation outcomes offer a foundation for researching the mechanisms of the developmental processes and pollination of the *P*. *orientalis* female cone.

**Figure 5:**
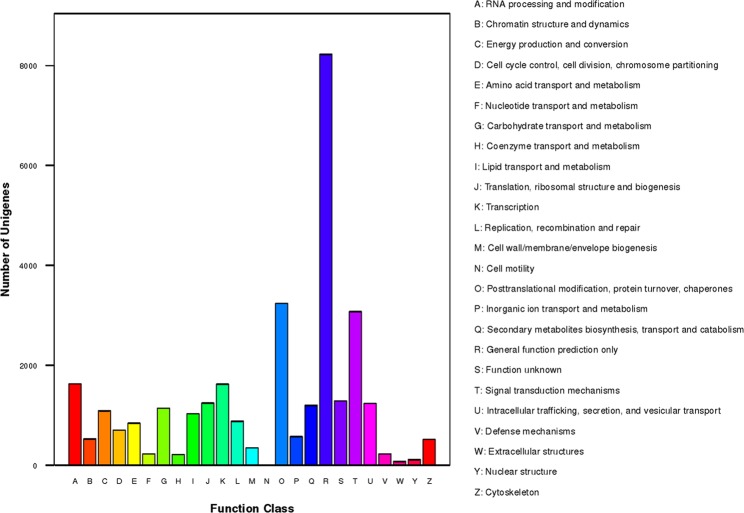
Histogram statistics of the KOG categorisations of *P*. *orientalis* unigenes. The abscissa represents the function clusters of KOG, and the ordinate represents the unigene number for a specific cluster.

To determine the possible functions, the total unigenes were aligned to sequences in the KOG from 7 eukaryotic complete genomes in the clusters of orthologues or paralogs. As a result, 18,800 (26.23%) unigenes were annotated on 36,523 proteins or protein domains of 3,823 KOG identities divided into 25 clusters (Supplementary Table S4). From the results of KOG categorisations (Fig. 5), it was clear that R (general function prediction only; 8,220, 11.47% of all unigenes) represented the most abundant cluster, followed by O (post-translational modification, protein turnover, and chaperones; 3,237, 4.52%), T (signal transduction mechanisms; 3,074, 4.29%), A (RNA processing and modification; 1,628, 2.27%), K (transcription; 1,621, 2.26%), S (function unknown; 1,283, 1.79%), J (translation, ribosomal structure and biogenesis; 1,243, 1.73%), and U (intracellular trafficking, secretion, and vesicular transport; 1,234, 1.72%).

### Annotation and categorisation of transcriptomic data by KEGG

**Figure 6:**
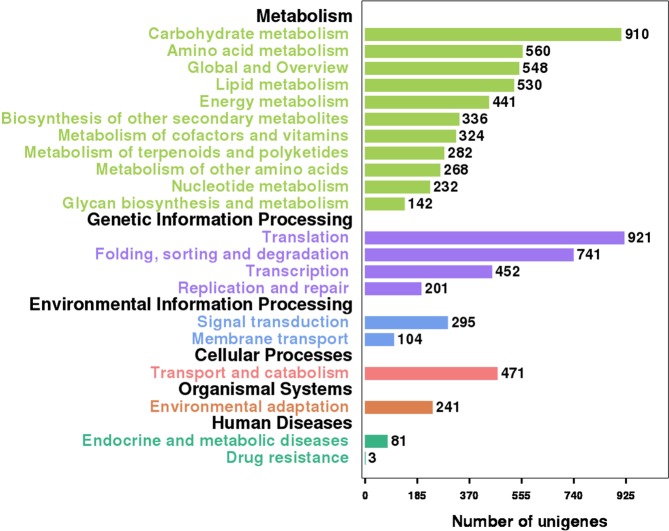
KEGG pathway categorisations of the identified unigenes. The abscissa represents the unigene number for a specific category, and the ordinate represents the categories of the KEGG pathways. Each colour shows a different category.

To analyse the active pathways in female cones development of *P*. *orientalis*, all 71,669 identified unigenes were analysed using the KEGG database, and 11,086 (15.47%) were assigned to the KO database and 6,007 (8.38%) to the pathway database that included 132 KEGG pathways of 6 main categories (Supplementary Table S5). The ten largest pathway groups were ribosome [ko03010, 351 unigenes (5.88% of assigned unigenes)], spliceosome [ko03040, 324 (5.42%)], carbon metabolism [ko01200, 318 (5.32%)], protein processing in endoplasmic reticulum [ko04141, 300 (5.02%)], biosynthesis of amino acids [ko01230, 266 (4.45%)], starch and sucrose metabolism [ko00500, 235 (3.93%)], endocytosis [ko04144, 228 (3.82%)], plant hormone signal transduction [ko04075, 225 (3.77%)], phenylpropanoid biosynthesis [ko00940, 201 (3.37%)] and RNA transport [ko03013, 190 (3.18%)] (Supplementary Table S5). Figure 6 shows the characteristics of the pathway categorisations depending on the KEGG pathway database. The largest category was metabolism (3163 unigenes), followed by genetic information processing (2131), cellular processes (471), environmental information processing (399) and organismal systems (241). These results indicate that metabolic and genetic information processing occurred actively during the pollination period, and these annotations of unigene descriptions and biological pathways represent available datasets for future investigations of the female cones of *P*. *orientalis*.

### Analyses of differential unigene expression during the development of female cone

To research the differences of unigene expression profiles in female cones of *P*. *orientalis* during the pollination period and to determine the critical unigenes responsible for the development of female cones, clean reads of the Befl (pre-pollination group) and Fl (pollination group) were assembled and aligned to reference data using SOAPaligner/soap2 and Trinity with the measurement of expression levels that were evaluated using RPKM. DEGs were identified based on |log_2_ FC| > 1 using FDR < 0.05. There are 16,710 DEGs were identified at the two development stages of female cone, composed of 4,963 upregulated unigenes and 11,747 downregulated unigenes at the pollination stage (Supplementary Fig. S2).

A PCA computed using the transcriptomic data allowed us to distinguish the pollination from the pre-pollination samples visually, with the first principal component in the x-axis explaining approximately 89.5% of the overall variance (Supplementary Fig. S3). Correlation analysis of replicas (Supplementary Figs S4 and S5) revealed good repeatability with all 6 Pearson correlations for the parallel libraries greater than 0.95. These analyses revealed very high uniformity of the biological replicates for the female cone samples collected at both developmental stages, whereas the separation among pre-pollination and pollination samples was very well marked.

GO and KEGG analyses were conducted within the DEGs to reveal the molecular events that occurred during the development of female cones and to determine the pathways that might respond to DEGs. Within the BP ontology, the upregulated or downregulated DEGs at the pollination stage were most often assigned to the GO terms: metabolic process (2,168), cellular process (1,943), single-organism process (1,624) and organic substance metabolic process (GO:0071704, 1,483). Within the CC and MF ontologies, the DEGs were annotated most frequently in the GO terms: catalytic activity (2,349), binding (1,950), cell (1,385), cell part (1,384), intracellular (GO:0005622, 1,265) and intracellular part (GO:0044424, 1,259) (Supplementary Table S6). Depending on the above analyses, the statements can be issued that the expression of these unigenes was altered substantially in the sexual mature female cones and that the unigenes might contribute to the normal development of female cones.

The most abundant pathways which might manage the responses of DEGs were starch and sucrose metabolism (ko00500, 95), phenylpropanoid biosynthesis (ko00940, 91), plant hormone signal transduction (ko04075, 76), protein processing in endoplasmic reticulum (ko04141, 64) and carbon metabolism (ko01200, 60). Pathways such as flavonoid biosynthesis (ko00941), biosynthesis of amino acids (ko01230), circadian rhythm - plant (ko04712), glutathione metabolism (ko00480), plant - pathogen interaction (ko04626) and pentose and glucuronate interconversions (ko00040) were also annotated (Supplementary Table S7). The outcomes indicate that these pathway unigenes are vital for the development of *P*. *orientalis* female cones.

GO enrichment analysis was conducted within the DEGs to determine the GO terms with significant enrichment (*Q*-value ≤ 0.05) in the developmental process of female cones. 141 GO terms of BP ontology were significantly enriched in commonly upregulated DEGs at the pollination stage, such as “cell cycle (GO:0007049)”, “regulation of DNA metabolic process (GO:0051052)”, “cell cycle process (GO:0022402)”, “cellular component organization (GO:0016043)”, “polysaccharide metabolic process (GO:0005976)” and “flavonoid metabolic process (GO:0009812)”. The commonly upregulated DEGs at the pollination stage were significantly enriched for 3 analogous terms of CC ontology, “chromosome (GO:0005694)”, “chromosomal part (GO:0044427)” and “chromatin (GO:0000785)”, followed by 11 terms, such as “extracellular region (GO:0005576)”, “non-membrane-bounded organelle (GO:0043228)” and “external encapsulating structure (GO:0030312)”. The commonly upregulated DEGs at the pollination stage were significantly enriched for 20 GO terms of MF ontology, containing “catalytic activity”, “oxidoreductase activity (GO:0016491)”, “hydrolase activity, acting on glycosyl bonds (GO:0016798)”, “transferase activity (GO:0016740)” and “lyase activity (GO:0016829)”. By contrast, the DEGs that were commonly downregulated at the pollination stage were significantly enriched only in 6 terms in the BP “response to stimulus (GO:0050896)” and “glycolipid biosynthetic process (GO:0009247)”, in the CC “lipid particle (GO:0005811)”, and in the MF “oxidoreductase activity”, “UDP-galactosyltransferase activity (GO:0035250)” and “galactosyltransferase activity (GO:0008378)” (Supplementary Table S8).These results suggest that the female cones of *P*. *orientalis* mobilize multiple functional aspects towards sexual maturity and through functional change of the expression profiles of several unigenes.

To ascertain the enriched pathways in female cones between the two stages, the DEGs were aligned to the biological pathways in KEGG using KOBAS, which were screened with a *Q*-value (corrected *p*-value subjected to the multiple hypothesis test; the smaller, the more remarkable) cut-off of 0.05. 17 biological pathways, e.g. “flavonoid biosynthesis”, “phenylpropanoid biosynthesis”, “starch and sucrose metabolism”, “circadian rhythm - plant”, “diterpenoid biosynthesis (ko00904)”, “betalain biosynthesis (ko00965)”, “DNA replication (ko03030)” and “monoterpenoid biosynthesis (ko00902)”, were significantly enriched in commonly upregulated DEGs. However, unigenes in 11 pathways, including “glutathione metabolism”, “zeatin biosynthesis (ko00908)”, “galactose metabolism (ko00052)” and “arachidonic acid metabolism (ko00590)”, were significantly enriched in commonly downregulated DEGs (Supplementary Table S7 and Fig. S6). The identification of the KEGG pathways that are significantly enriched is important for elucidating the mechanism underlying the developmental process of *P*. *orientalis* female cone.

As essential upstream regulators, Transcription factors (TFs) function substantially to prompt full-blown cones at the reproductive growth stage of plants. Unigenes encoding proteins were mapped to Plant TFdb (http://planttfdb.cbi.pku.edu.cn/) by BLASTp analysis to predict TF families. In this study, 1106 TF unigenes were identified and classified into 55 families, in which *ERF* was the largest family, followed by *bHLH*, *MYB*-related and *MYB*. Thereinto, 361 TF unigenes were differentially expressed between the developmental processes of female cones, and these TF unigenes were categorised in 45 families, in which the most abundant family of TF unigenes was also *ERF* (58, 16.07%), followed by *MYB* (46, 12.74%), *bHLH* (29, 8.03%), *MYB*-related (24, 6.65%), *C2H2* (20, 5.54%), *NAC* (17, 4.71%), *WRKY* (16, 4.43%), *B3* (10, 2.77%), *HD-ZIP* (10, 2.77%), *MIKC* (10, 2.77%) and *bZIP* (9, 2.49%)(Supplementary Table S9).

### qRT-PCR validation of differentially expressed unigenes

**Figure 7:**
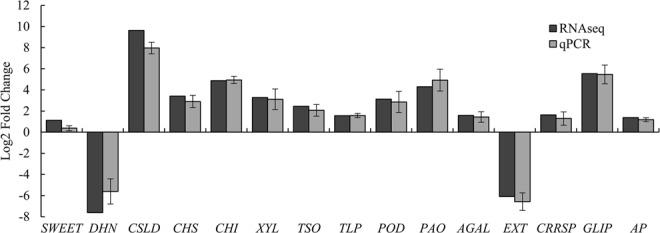
The changes in expression levels of unigenes confirmed by qRT-PCR. Abbreviations for genes: *SWEET*, bidirectional sugar transporter SWEET1-like; *TSO*, CRC domain-containing protein TSO1-like; *CSLD*, cellulose synthase-like D3-1; *CHS*, chalcone synthase; *CHI*, class II chitinase; *XYL*, Beta-xylosidase; *DHN*, dehydrin 1; *EXT*, extensin-2; *POD*, peroxidase; *PAO*, polyamine oxidase; *AGAL*, alpha-galactosidase; *TLP*, thaumatin-like protein L1; *CRRSP*, cysteine-rich repeat secretory protein 55; *GLIP*, GDSL esterase/lipase; and *AP*, aspartyl protease family protein. The expression change of the unigenes are represented as log_2_ FC of Fl/Befl and are exhibited on the ordinate. Black bars are RNA-seq results; gray bars are qRT-PCR results. The UBC genes were used as reference genes. The qRT-PCR values are exhibited as the mean ± SD.

To verify the RNA-Seq data, 15 candidate DEGs were chosen and evaluated by qRT-PCR. Thirteen unigenes, namely, bidirectional sugar transporter SWEET1-like (*SWEET*), CRC domain-containing protein TSO1-like (*TSO*), cellulose synthase-like D3-1 (*CSLD*), chalcone synthase (*CHS*), class II chitinase (*CHI*), Beta-xylosidase (*XYL*), peroxidase (*POD*), polyamine oxidase (*PAO*), alpha-galactosidase (*AGAL*), thaumatin-like protein L1 (*TLP*), cysteine-rich repeat secretory protein 55 (*CRRSP*), GDSL esterase/lipase (*GLIP*) and aspartyl protease family protein (*AP*), were identified as upregulated at the pollination stage, and two unigenes, including dehydrin 1 (*DHN*) and extensin-2 (*EXT*), were identified as downregulated at the pollination stage (Fig. 7). The expression profiles determined by qRT-PCR were roughly in accordance with the differences in expression levels of the unigenes, evaluated by the RNA-Seq, with the same tendency. The primer information, RNA-Seq outcomes and qRT-PCR values are shown in Supplementary Table S10. The qRT-PCR outcomes were approximately same as those examined by RNA-Seq, supporting the strong dependability of the RNA-Seq results. The differences regarding ratio could likely be ascribed to the discrepancies of algorithms and sensitivities between the two methods.

## Discussion

### Sequencing and unigene annotation of *P*. *orientalis* female cones

*P*. *orientalis* is an important tree that is widely used for afforestation, landscaping, wood and medical ingredients, and it is widespread in the northern regions of China, especially in the Loess Plateau. However, less is known about the molecular mechanism underlying its reproduction. Transcriptomic analyses of the pollination process are a valid way of evaluating the unigenes, functions and processes that are vital and liable for growth and reproductive events in female cones. In this work, abundant unigenes of *P*. *orientalis* (71,669) were sequenced by Illumina HiSeq 4000 (Table 3). The average length of the unigenes was 908.36 bp, and the N50 length was 1,621 bp. These outcomes are close to the newly published transcriptomic researches of other gymnosperms, e.g. *Ginkgo biloba*^27^, *Pinus bungeana*^28^ and *Sabina chinensis*^29^.

In this study, 17,403 (24.28%) and 11,086 (15.47%) unigenes were enriched in GO terms and the KEGG database, respectively. Only some of the unigenes could be annotated. The lack of gymnosperm sequence data available for gene identification was a limiting factor in the transcriptome analyses. As informative and dependable markers in organism study, SSRs are more polymorphic than the large proportion of other marker systems. 2,840 SSRs of 154 motif types were identified in 2,477 unigenes of this transcriptome. The results are similar to some previous researches^22,28,29^. The SSRs will provide new fingerprinting for taxonomic and phylogenetic contrasts. They also can serve as a mapping means in many other species. Our results will highly improve the present knowledge of the *P*. *orientalis* and will be conducive to the abundance of sequence resources for studying Cupressaceae and other conifer species. These data will help define the mechanisms of flower tissue specificity in a non-model plant organism. These datasets will also promote research aimed at the identification of novel genes related to the reproductive processes of woody plants.

### Functional enrichment analyses of DEGs during developmental processes

To be more specific, cone samples of *P*. *orientalis* were collected as succinctly as possible without any unnecessary operations, and the cone samples were maintained in consistent states and conditions between pre-pollination and pollination except for the developmental stages when the cones were sliced. There are numerous differentially expressed unigenes were identified between the two growth periods. Comparative transcriptome profiling of *P*. *orientalis* cones at the pre-pollination and pollination stages identified 16,710 DEGs. Among them, 4,963 were upregulated, and 11,747 were downregulated, suggesting distinct molecular mechanisms at different growth stages. The outcomes indicate that organisms attempt to change their gene expression activity to ensure the success of the reproductive event. More genes were downregulated than upregulated in this study, likely as a consequence of the pre-pollination female cones undergoing invisible changes and accumulation to prepare for development and pollination. The results are similar to other studies focused on the regulation of flower formation in other plants, such as *Camellia sinensis*^1^, *Arabidopsis thaliana*^5^ and *Carya cathayensis*^4^.

The DEGs functional enrichment analyses will give insight into the genetic mechanisms by which *P*. *orientalis* accomplishes cone growth and pollination. Functional researches have showed an intricate genetic system which control the changing of flower development^30^. Depending on the GO enrichment analysis of the DEGs, commonly upregulated DEGs were significantly enriched in the developmental processes of integrated reproductive function [i.e., reproduction (GO:0000003), multicellular organismal reproductive process (GO:0048609), single organism reproductive process (GO:0044702) and multicellular organism reproduction (GO:0032504)], the generation of macrogametocyte [i.e., meiotic nuclear division (GO:0007126), meiotic cell cycle (GO:0051321), meiotic cell cycle process (GO:1903046), chromosome organization involved in meiosis and gametophyte development (GO:0070192)] and the development of the megastrobilus [i.e., flower development (GO:0009908), floral organ development (GO:0048437)] (Supplementary Table S8). This phenomenon is similar to *Chimonanthus praecox*^6^, *Cymbidium sinense*^25^ and *Carya cathayensis*^4^. The active upregulated enzymes that pertain to the above GO terms will guarantee successful sexual maturity and timely receptiveness of the female cones.

The cones consume a great deal of energy to meet the reproductive growth needs for the synthesis and metabolism of massive substances during the developmental process, with morphological changes in colour, size increases and bract opening. For example, starch can be found to gather within nucellus of several gymnosperm plants before pollination^10,12^, and it decreases at the pollination stage^11^. In this study, the upregulated GO terms in BP ontology such as carbohydrate catabolic process (GO:0016052), polysaccharide metabolic process, carbohydrate metabolic process (GO:0005975), very long-chain fatty acid metabolic process (GO:0000038), carbohydrate derivative catabolic process (GO:1901136), cellulose metabolic process (GO:0030243), polysaccharide catabolic process (GO:0000272), beta-glucan metabolic process (GO:0051273), glucan metabolic process (GO:0044042), cell wall polysaccharide metabolic process (GO:0010383), lipid metabolic process (GO:0006629), cell wall macromolecule metabolic process (GO:0044036) and xylan metabolic process (GO:0045491) were significantly enriched at the pollination stage. These results showed that energy-based pathways were active and synergetic during cone development. In these terms, a considerable scale of the unigenes encoding proteins are related to cell wall catabolism, such as isoforms of beta-glucanase and glucanase-like protein (GLP)^31^. The enhancement of the activity of glucosidase (GLU), one of which breaks down polysaccharides and glucoside to sugar, and GALs, which metabolize a variety of polysaccharides^32^, sustains energy expenditure for the reproductive activities of female cones. Other upregulated categories in MFs, such as beta-glucosidase activity (GO:0008422), glucosidase activity (GO:0015926), hydrolase activity (GO:0016787) [particularly, acting on glycosyl bonds and hydrolysing O-glycosyl compounds (GO:0004553)], transferase activity[(particularly, transferring glycosyl groups (GO:0016757) and hexosyl groups (GO:0016758)], carbon-oxygen lyase activity (GO:0016835) [(particularly, acting on polysaccharides (GO:0016837)] and oxidoreductase activity, were also significantly enriched at the pollination stage. Considerable proteins encoded by the above unigenes participated in enriched pathways associated with the energy supply, e.g. “starch and sucrose metabolism”. These active enzymes together will ensure a sufficient energy supply for the development of female cones in *P*. *orientalis*.

Flavonoids are formed from the basic core structure of flavane scaffolds, which are a widely existing signature category of extensively decorated secondary metabolites^33^. They are vital in human health and plant physiological activity. There are diversified flavonoids in female cones of *P*. *orientalis*^21^. They can change the colour of female cones from green to yellow during the developmental process, preventing UV radiation damage^34^. They can also resist bacterial and herbivore attack and contribute to cone fecundity and pollen germination^35^. In this study, 4 upregulated enriched pathways were related to flavonoid biosynthesis, among which the central pathway was “flavonoid biosynthesis”, which derives from “phenylpropanoid biosynthesis” and is linked to “flavone and flavonol biosynthesis (ko00944)” and “anthocyanin biosynthesis (ko00942)”. Flavonoid biosynthesis is highly active at the pollination stage because the most pivotal rate-limiting enzyme genes are significantly upregulated, including 15 *CHS*, 1 chalcone isomerase, 3 flavanone 3-dioxygenase (*F3H*), 3 flavonoid 3′,5′-hydroxylase (*F3'5′H*), 6 dihydroflavonol 4-reductase genes (*DFR*). The outcomes are comparable to those examined in the ovule of *Ginkgo biloba*^36^, female buds of *Metasequoia glyptostroboides*^37^ and female flowers of *Humulus lupulus*^38^. These results demonstrate that flavonoids are indispensable for female reproductive organs at the pollination stage for plants to activate and manage enzymes and pathways by TFs.

A total of 1106 TFs from 55 families were identified, and 361 TFs from 45 families showed significant differences between the two stages of female cones. These results are similar to previous studies^22^. TFs play an important role in almost all biological processes^39^. Therefore, the determination of the TFs provides a broader perspective for exploring the complex processes of the regulation of the female cone development in *P*. *orientalis*. Although the functional studies of TFs have accumulated in many species, the regulatory role of the TFs in the development of female cones of *P*. *orientalis* needs to be further studied.

### Preliminary molecular mechanism analyses of the secretion and function of the pollination drop

#### The unigenes related to ovule secretion for the pollination drop

As an indispensable part of pollination mechanism of female cone, the pollination drops of living gymnosperm lineages are involuted ovular secretions from nucellar tip cells. Some phenomena have been observed in nucellar cells, for example, the fluctuating cell membranes, agminate high-density electrons, the extensive and active Golgi apparatus and endoplasmic reticulum during secretion, and the accumulation of starch grains before secretion^10–12,40,41^. These phenomena indicate that secretion of the pollination drop may be related to transmembrane transport, and the transport efficiency of water and small solutes in plants is closely related to aquaporins (AQPs)^42^. As channel proteins, AQPs belong to major intrinsic protein family, including TIPs, PIPs and SIPs which facilitate the diffusion and transport of water and amino acids etc. across tonoplast and cell membrane^43^. In the transcriptome, 29 unigenes were annotated to be associated with *AQPs*, of which 4 *TIPs*, 4 *PIPs* and 1 *SIP* were significantly upregulated, especially all upregulated *TIPs*, and most of them were assigned to water transmembrane transporter activity (GO: 0005372). So these AQPs may participate in the secretory process of the pollination drop. Moreover, the above phenomena are similar to those observed for nectar secretion^44^, indicating that secretion of the pollination drop and nectar may share a similar form and process, even mechanism. However, many genes that play important roles in nectar secretion, such as *NEC1*, *CYP86B1*, *Nectarin-I* and *Nectarin-III*, could not be detected in this transcriptome, other than one insignificant *SWEET9*^45^ annotated by Swiss-Prot. Thus, the molecular mechanisms responsible for secretion of the pollination drop and nectar may differ. One secretory carrier-associated membrane protein 2 (SCAMP2) and one SCAMP3, involved in cell membrane trafficking and exocytosis^46^, may participate in the transport and secretion of the pollination drop. Three significantly upregulated unigenes were found to be annotated bidirectional sugar transporter *SWEET* protein family, encoding enzymes that function in the efflux of sugar across the plasma membrane^47^, zealously participating in plant reproductive development^48^, which may be related to secretion of the pollination drop. Furthermore, the alterations in the osmotic potential of pollination drop are thought to stimulate the movement of water^17^. As osmotic regulators^45^, sugars can be proposed to regulate drop formation in gymnosperms^8^. Moreover, the carbohydrate composition of the pollination drop is mainly composed of fructose with reducibility^8,11,12^, rather than the more stable and common sucrose, which may cater to the needs of pollen^49^ and maintain normal germination of the target pollen. Annotated by seventeen unigenes, invertases (INVs) may be related to sucrose declining and fructose and glucose rising in the pollination drop^50^. Two annotated sorbitol dehydrogenases, related to the accumulation of the fructose, may be involved in formation of the pollination drop. The pectinesterases (PMEs) and PERs, encoded by upregulated unigenes, may promote the accumulation of extracellular matrix by modulating the relaxation of surface cells^51^, which may partake in pollination drop secretion.

In addition, the encoded antifreeze protein GLPs and thaumatin-like proteins (TLPs), with dual adjustment functions^52^, may be related to the stable state of the pollination drop in the early spring nights. The surface of integuments with hydrophobic keratin can avoid rain to wash away the pollination drops and improve the pollination efficiency^7,10^, which could be attributed to the significantly enriched pathway of “cutin, suberine and wax biosynthesis (ko00073)”. The encoded lipid transfer proteins (LTPs) are also involved in wax or cutin deposition^53^. Our research and speculation provide hypotheses and demonstrate a need for further investigations.

#### The programmed cell death (PCD) of nucellus with generation of pollination drop

Before the pollination drop appears, a part of the nucellus cells will degrade in the form of PCD in *P*. *orientalis*^40^, similar to many species^7,11,12,41^. Some researchers consider the degradation products to induce or participate in the formation of pollination drops^41^. The pollination drop contains multiple carbohydrates, amino acids, proteins and other components^8,11,12,17,19,41^, which may be part of the catabolites of PCD^8,12^. To verify the hypothesis, proteins were identified in the pollination drop of *P*. *orientalis*. There are three different proteins were determined, including ribulose 1,5-bisphosphate carboxylase, partial (chloroplast), photosystem II subunit L (chloroplast) and sabinene synthase, which were clearly not secreted proteins (Supplementary Table S11). The results support the claim above. In this transcriptome, 103 unigenes were annotated with PCD according to the GO BP terms. Among them, a Cys proteinase vacuolar processing enzyme (VPE) which is localized within the region with heavy electron around cell walls of the cells undergoing cell death^54^, was confirmed as the executor of plant PCD by processing many vacuolar proteases and hydrolases related to PCD^55^. The unigene annotated as nitric oxide (NO) synthase-interacting protein supported the viewpoint that NO is also a key factor in the execution of PCD^56^. The increasing levels of reactive oxygen species (ROS) are used as signal mediators, which are also included in the mechanism of PCD induction^57^. Thus, numerous unigenes related to the generation and transfer of ROS may participate in PCD. Annotated regulatory genes such as *LSD1*, *EDS1* and lipase-like *PAD4*, the protein products of which function upstream of ROS production, mediate PCD initiation, together with SAG101^58,59^. PCD can be induced by metal (copper and zinc) which induced generation of ROS^60^, and metal homeostasis is related to metallothionein type 3, copper transport protein ATX1 and molybdopterin biosynthesis protein CNX1^61^, which are all encoded by upregulated unigenes during the pollination stage. The significantly upregulated PAOs conduce directly to the load of H_2_O_2_ in the apoplast, that is due to catabolizing polyamines (PAs) such as spermidine^62^. PAs derived from biosynthesis catalysed by spermidine synthase and arginine decarboxylase are extruded via the polyamine transporter and cationic amino acid transporter to the apoplast, where they are then targeted by PAO, which further elevates the ROS concentration^63^. One of the products of the subsequent reactions is H_2_O_2_, which may also induce PCD^64^. The respiratory burst oxidase homolog (*RBOH*), which is also involved in the generation of ROS, and *BAX* inhibitor1, a modulator of PCD, together are treated as PCD marker genes^65^. PCD can also be triggered by NBS-LRR resistance proteins such as RPS4^66^. The encoded enzymes associated with cell wall catabolism, such as GLPs and GLUs^31^, may be directly involved in PCD. Likewise, tryptophan synthase, cysteine protease, serine hydrolase, anamorsin homologue 1, arabinogalactan proteins (AGPs), anthranilate synthase, male sterility, transcription factor TDR and AMS, all encoded by unigenes, are thought to be putative regulators or controllers of PCD in different species and tissues^67,68^. These annotated unigenes may synergistically facilitate the normal and timely PCD of nucellar tissue. However, many unigenes, directly or indirectly related to PCD, have not been discovered. Therefore, PCD of the nucellar cells can be predicted to represent a multi-gene synergistic process that may be managed by a large pathway in *P*. *orientalis*.

#### The relationship between the pollination drop and pollination

The main functions of pollination drop are to capture pollens and transport the pollens into nucellus. Pollination drop may play several roles to ensure pollination successful with this process, and the components of pollinating drop contribute vitally to the achievement of these roles. The pollination drop contains carbohydrates, amino acids and other components^8,11,12,17,19,41^, which provide nutrition to the pollen and help target pollen germination. Large differences in the compositions and contents of heterogenous pollination drops may provide a suitable environment for homologous pollens and affect the adaptability of heterologous pollens^69^. These operations depend on the precise efforts of a number of enzymes and TFs encoded by the significantly upregulated metabolism-related unigenes in the ovule at the pollination stage. Especially, the encoded proteins, INVs, APs, serine carboxypeptidases (CBPs) and subtilisin-like proteases (SBTs), may provide nutrition for pollen germination^19,50^, and glycosyl hydrolases, xylosidases, GLPs, GALs, PERs, glucan 1,3-β-glucosidases (BGLs) and AGPs may be beneficial for pollen tube elongation^19,32,50^, which all are detected in the pollination drop^18^. Moreover, glutamate decarboxylase, chemocyanin, PMEs, LRR receptor-like serine/threonine-protein kinase, LTPs and AGPs, encoded by upregulated unigenes, may function in guidance to control polarized pollen tube growth to the micropyle^16,51,53^. Additionally, PERs, BGLs, CHIs and TLPs in the pollination drop can lyse the fungal cell wall to achieve an antibacterial effect^17,19,50^, and SBTs, APs, and CBPs may transport the pathogen defence signal^19,50^. These encoded active enzymes and compounds together might provide a suitable environment for the target pollen in the pollination drop.

When active conspecific pollens land on the pollination drop, the pollination drop can recognize the pollen and accelerate withdrawal in *P*. *orientalis*^14,15^, similar to many other species^9,11,13^. The functional proteins in the pollination drop may be charged with the most important responsibility for the identification of pollen. Although the molecular mechanisms of plant recognition and the immune response of pathogenic microorganisms have been extensively studied^70^, there few investigations have addressed the recognition and response of the pollination drop to pollen. In this study some identified unigenes may be related to the recognition based on RNA-Seq data. As the major biological recognition proteins, glycoproteins contain various sugar chains with a wealth of structural information, which are often recognition sites for receptors and enzymes^71^. The hydroxyproline-rich glycoproteins and AGPs, especially fasciclin-like AGPs, which are encoded by the significantly upregulated unigenes at the pollination stage and discovered in the pollination drops of several species^16,18^, may participate in the recognition. INVs, especially alkaline/neutral invertase (CINV), can distinguish homologous pollens while excluding heterologous pollens by modulating the osmotic potential of the pollination drop^72^, together with PERs^50^. The *LTPs* genes encoding the proteins containing a secretory signal peptide, which mediate cell wall loosening *in vitro* and are detected in the pollination drop^18^, may participate in the interaction with the pollen surface. Some annotated serine/threonine-protein kinases (*SRKs*) genes encoding polymorphic S-locus receptor kinases, with homologs in the pollination drop of cycads^18^, control pollen recognition on the papillar surface of Brassicaceae^73^. The functioning pathway of SRK may be involved in the Armadillo repeat-containing protein (ARC) and *EXO70A1*, annotated by upregulated unigenes, which may also participate in the secretion of the pollination drop and the hydration of pollens^74^. As metabolism-related enzymes, oxidases can generate ROS intermediates to recognize pollens and to dissolve heterologous incompatible pollens in the pollination drop^19,50^. However, the molecular mechanisms of these annotated enzymes require further research to clarify their role in gymnosperm pollination, as most research has focused on angiosperms, especially self-incompatibility. Additionally, the genes, associated with secondary metabolites that perform the recognition function in roots^75^, have not been detected in the transcriptome, so the reproductive organ is likely to possess recognition mechanisms that differ from vegetative organs. Nevertheless, research remains scarce. Scholars can explore this field by using research methods and molecular mechanisms of interactions between plants and pathogenic microorganisms for reference.

### Glimpse of the unigenes in the female cone related to the flowering process

Flowering is controlled by complex networks that involve multiple signalling pathways, as described for the female cones of gymnosperms. Numerous genes participating in the flowering process of angiosperms (principally) could be annotated in the female cone of *P*. *orientalis*. For example, *STERILE APETALA* (*SAP*), *LEAFY* (*LFY*) and its orthologue *NEEDLY*, together with the floral homeotic genes *AGAMOUS* (*AG*) and *APETALA2* (*AP2*), all encode TFs that promote and maintain early floral meristem identity. The C class gene *AG*, which is upregulated by LFY and downregulated by A class cadastral proteins AP2 and SAP, is required for normal development of the reproductive organ^3^. Additionally, SAP interacts with AP2 to ensure the normal development of the ovule. Annotated *WUSCHEL* (*WUS*) and its homeoboxes encoding TFs that are downregulated by ULTRAPETALA 1 play central roles during flowering, such as activating the expression of *AG* and modulating one important step in ovule development^76^. The stable presence of *WUS* demonstrates that the duties of *WUS* may be conservative across the spermatophytes. The *UNUSUAL FLORAL ORGANS* (*UFO*) unigene, encoding a part of the ASK-cullin-F-box E3 ubiquitin ligase complexes, is needed for several biological processes in the developing flower, covering the appropriate patterns and characteristics of the petal^77^. Many annotated *AGL* (*MADS*-box) unigenes encode TFs that are essential for the development of reproductive organ^39^, such as the control of female gametophyte development (*AGL11* and *AGL61*), control of floral meristem identity and subsequent transition (*AGL8* and *AGL24*), mediation of *LFY* expression linking floral induction and floral development (*SOC1*, *AGL8* and *AGL24*), negative regulation of flowering through the photoperiodic pathway (*AGL15* and *AGL18*), mediation of floral transition and transduction of RLK-mediated signalling (*AGL24*). The annotated *GIGANTEA* activates TFs encoded by the zinc finger gene *CONSTANS*, which may mediate the circadian clock and control of flowering^78^. Moreover, the encoded B-sister MADS-box TFs may also have a positive function in ovule development^79^. The female cone utilizes a variety of mechanisms to ensure the normal progress of pollination during development, including the induction of various biochemical and physiological reactions to perfect itself. However, many functional genes and pathways that mainly act in the flowers of angiosperms cannot be annotated in the transcriptome of female cones of *P*. *orientalis*, such as *FLOWERING LOCUS C*, *TERMINAL FLOWER*, *PISTILLATA*, *CAULIFLOWER*, *FLOWERING LOCUS T*, *AP1*, *AP3*, *DAL11* and *DAL13*^39^. The autonomous pathway, regulating floral transition in angiosperms, is not detected. *LURE 1*, a female attractant for pollen signal perception in angiosperms, is also not annotated. Thus, these floral protein-protein interaction patterns of angiosperms are highly distinct from those in gymnosperms.

This work provides primary insight into DEGs during the developmental process of the female cone in *P*. *orientalis*. The functional validation of the pollination transcripts characterized herein will improve knowledge regarding the pollination biology in gymnosperms and research to further investigate the function of unannotated genes to clarify the reproductive mechanism.

## Conclusion

Pollination progression and the underlying mechanism in *P*. *orientalis* has been previously overlooked, considering its widespread distribution and considerable practical, ecological, and economical benefits. In this work, high-throughput transcriptomic data were used to evaluate the unigene expression profiles of the female cone in *P*. *orientalis*. We explored the development and pollination mechanism of the female cone and performed a comprehensive analysis of related genes. While DEGs encoding functional and regulatory proteins were detected to participate abundantly in reproduction, flavonoids, and the energy supply, many TFs were also determined. The mechanism of secretion and the function of the pollination drop, an important secretion at the pollination stage, was dissected with emphasis. These unigenes are helpful to investigate the pollination mechanisms of gymnosperms, and much of the genetic data provided the basis for the further elucidation of the reproductive mechanism from the perspective of genetics and molecular biology. These findings represent a valuable resource for future investigations.

## Supplementary information

Supplementary Information

Supplementary Table S1

Supplementary Table S2

Supplementary Table S3

Supplementary Table S4

Supplementary Table S5

Supplementary Table S6

Supplementary Table S7

Supplementary Table S8

Supplementary Table S9

## Data Availability

The sequencing raw data of this article have been deposited in a SRA database at the NCBI [https://www.ncbi.nlm.nih.gov/sra/SRP151174].
