# Author Correction: *De novo* assembly of the *Platycladus orientalis* (L.) Franco transcriptome provides insight into the development and pollination mechanism of female cone based on RNA-Seq data

**DOI:** 10.1038/s41598-019-53777-z

**Published:** 2019-11-15

**Authors:** Wei Zhou, Qi Chen, Xiao-Bing Wang, Tyler O. Hughes, Jian-Jun Liu, Xin Zhang

**Affiliations:** 1 College of Landscape Architecture and Arts, Northwest A&F University, Yangling, Shaanxi P.R. China; 2 School of Life Science and Technology, Xinxiang University, Xinxiang, Henan P.R. China; 3 Department of Biology, The Pennsylvania State University, University Park, PA 16802 USA; 4 Key Laboratory of Silviculture on the Loess Plateau State Forestry Administration, College of Forestry, Northwest A&F University, Yangling, P.R. China

Correction to: *Scientific Reports* 10.1038/s41598-019-46696-6, published online 15 July 2019

The original version of this Article contained an error in the name of the author Tyler O. Hughes, which was incorrectly given as Tyler Hughes.

Additionally, in the original version of this Article, Affiliation 3 was incorrectly given as ‘Department of Biology, Penn State University, Philadelphia, PA, USA’. The correct affiliation is listed below.

Department of Biology, The Pennsylvania State University, University Park, PA 16802, USA

This has now been corrected in the PDF and HTML versions of the Article, and in the accompanying Supplementary Information file.

