# Supplementary material for: De novo assembly of the Platycladus orientalis (L.) Franco transcriptome provides insight into the development and pollination mechanism of female cone based on RNA-Seq data
Source: Sci Rep. 2019 Jul 15;9:10191. doi: 10.1038/s41598-019-46696-6 (PMC6629706; doi:10.1038/s41598-019-46696-6)
Supplement: Supplementary file 1 — Supplementary Information [file 41598_2019_46696_MOESM1_ESM.doc]

***De novo* assembly of the *Platycladus orientalis* (L.) Franco** **transcriptome provides insight into** **the development and pollination mechanism of female cone based on RNA-Seq data**

**Wei Zhou^1†^, Qi Chen^1†^, Xiao-Bing Wang^2^, Tyler** **O. Hughes^3^, Jian-Jun Liu^1*^, Xin Zhang^4*^**

^1^College of Landscape Architecture and Arts, Northwest A&F University, Yangling, Shaanxi, P.R. China
^2^School of Life Science and Technology, Xinxiang University, Xinxiang, Henan, P.R. China
^3^Department of Biology, The Pennsylvania State University, University Park, PA 16802, USA
^4^Key Laboratory of Silviculture on the Loess Plateau State Forestry Administration, College of Forestry, Northwest A&F University, Yangling, P.R. China

^†^These authors have contributed equally to this work.

*** Correspondence:**Jian-Jun Liu, [ljj@nwsuaf.edu.cn](mailto:ljj@nwsuaf.edu.cn)
Xin Zhang, [xin.zhang@nwsuaf.edu.cn](mailto:xin.zhang@nwsuaf.edu.cn)

**Supplementary Information**

**Supplementary Figures**


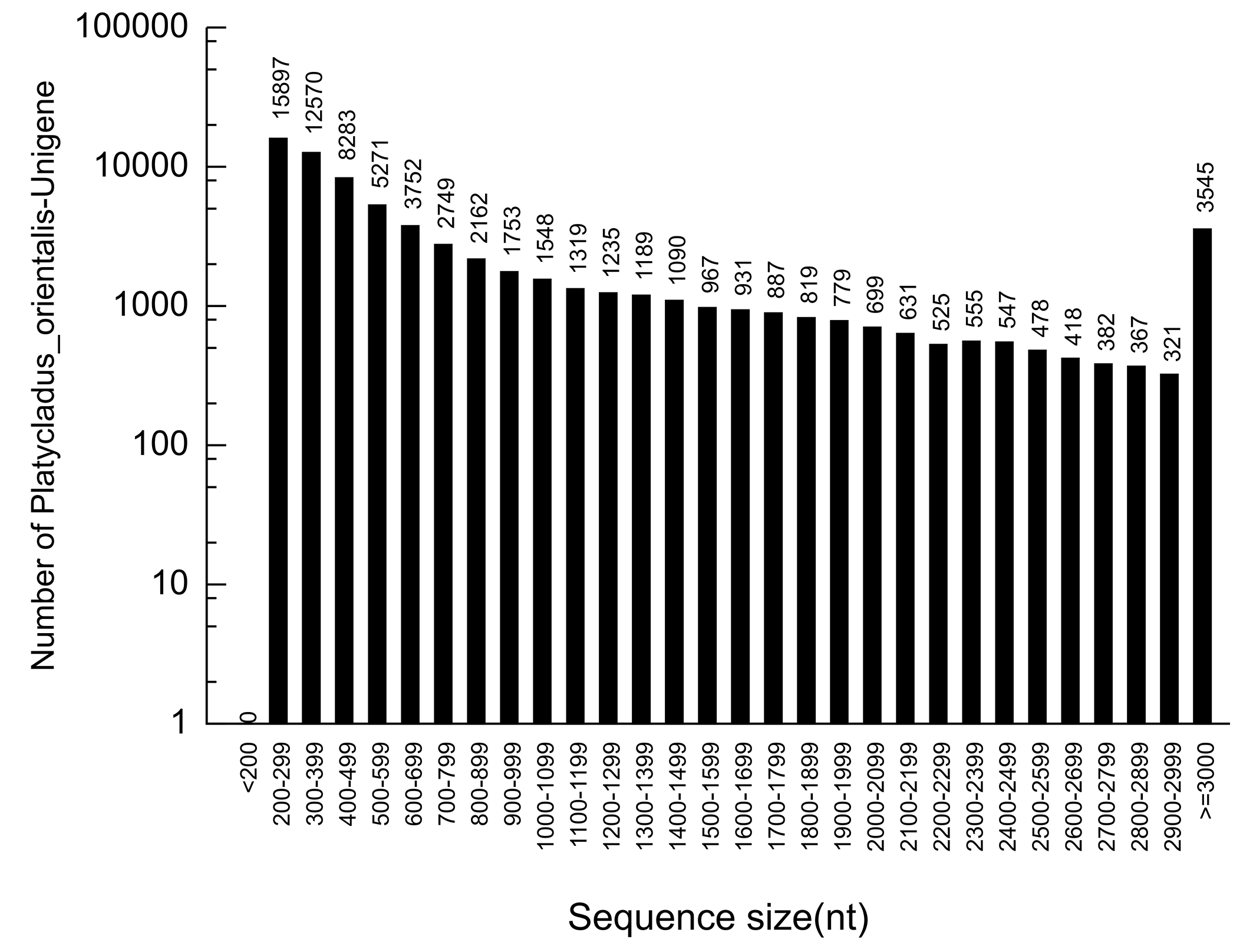


**Supplementary Figure S1** Length distribution of assembled unigenes.


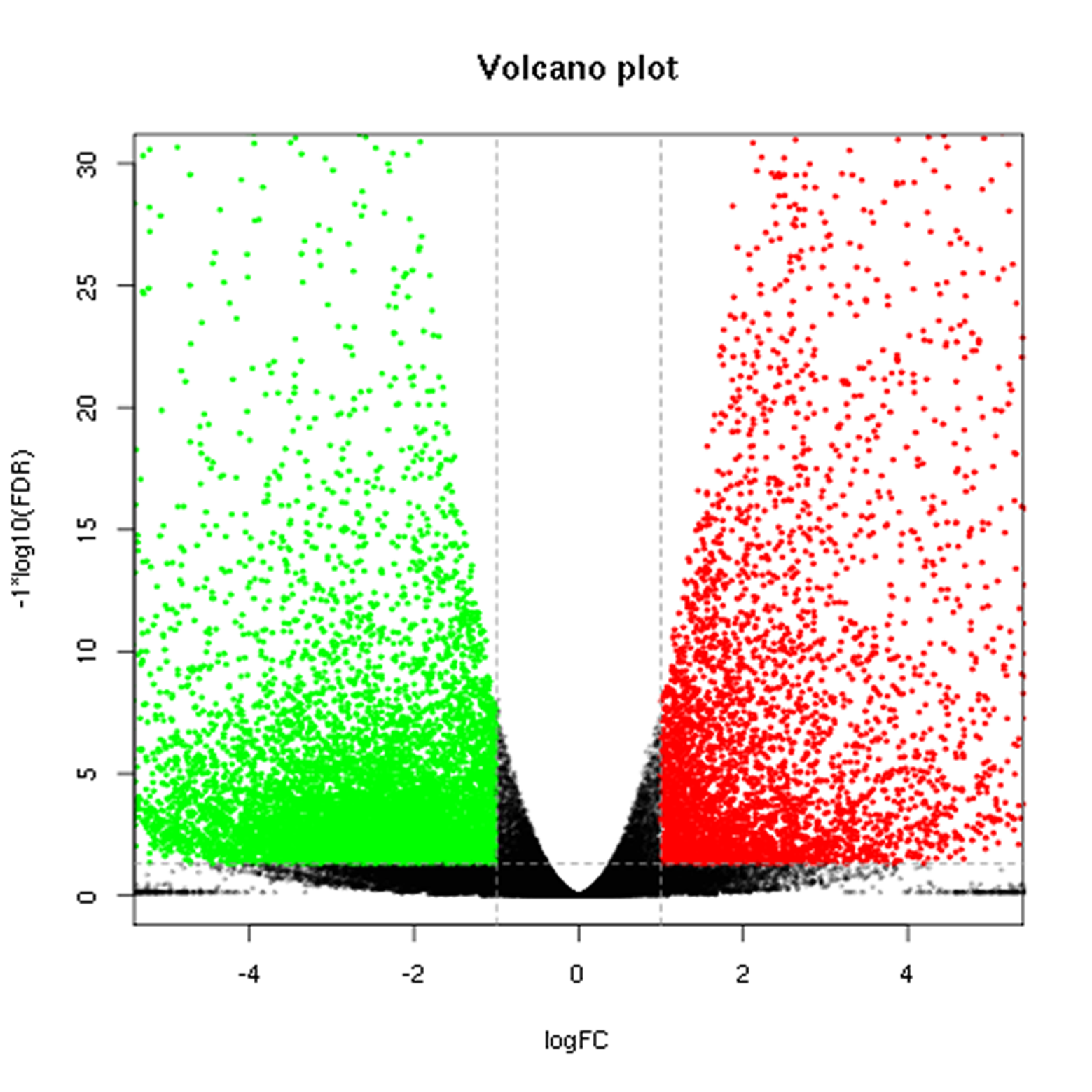


**Supplementary Figure S2** Volcano plot of differentially expressed genes between Befl and Fl. The x-axis indicates log-fold expression change between 2 groups, and the y-axis indicates -log10 of FDR between 2 groups. Red spots represented up-regulated genes (expression level of Fl vs Befl with a fold change greater than 2 and FDR<0.05), and green spots indicated down-regulated genes. Black spots represented genes that did not show obvious changes between 2 groups.


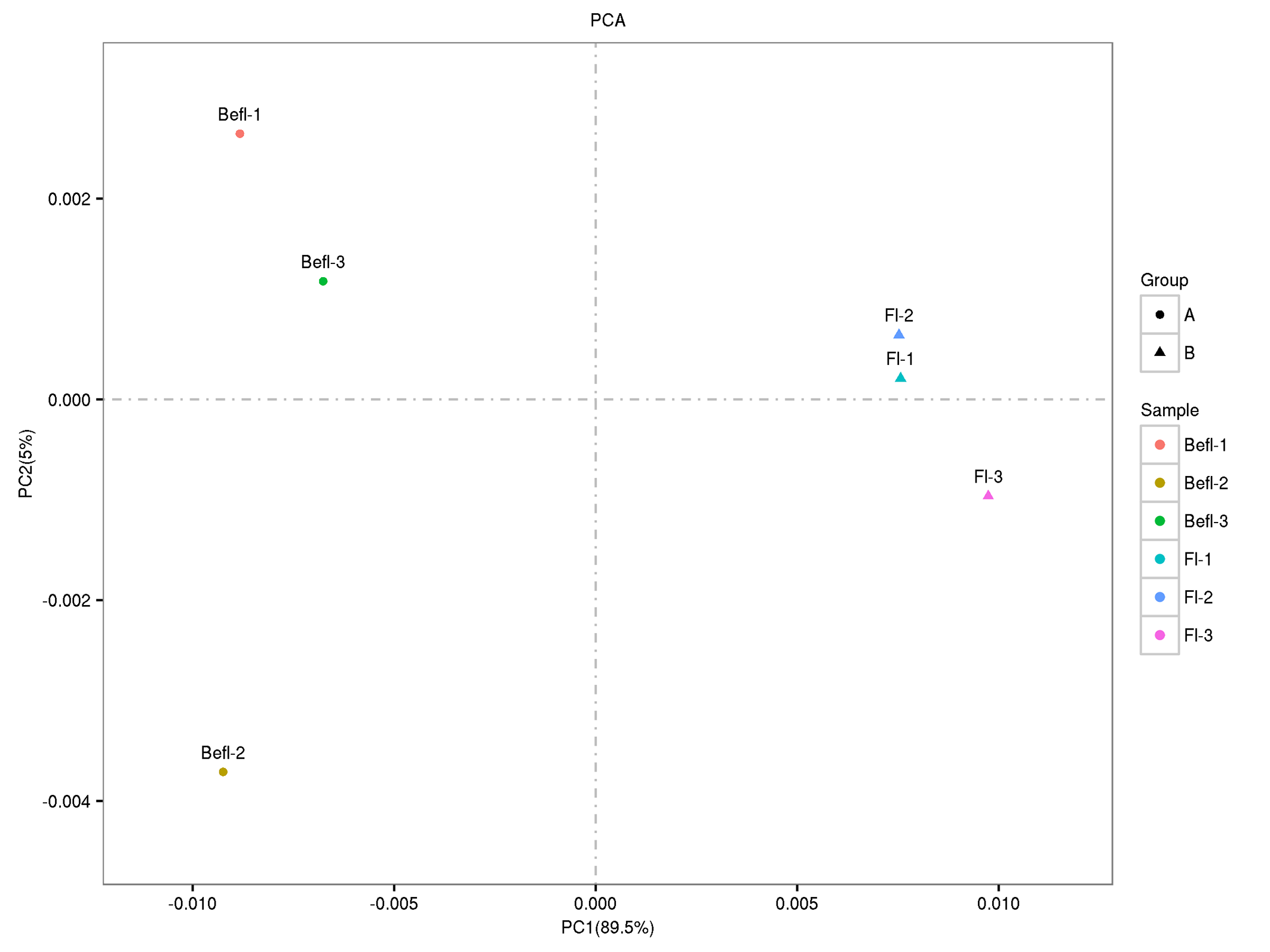


**Supplementary Figure S3** Principal component analysis (PCA) of gene expression in all samples of female cones. Befl1-3 indicate 3 replicates of female cones at pre-pollination stage; Fl1-3 indicate 3 replicates of female cones at pollination stage. The percentage variation explained by the two axes is approximately 94.5%, including the first principal component in x-axis explained about 89.5%.


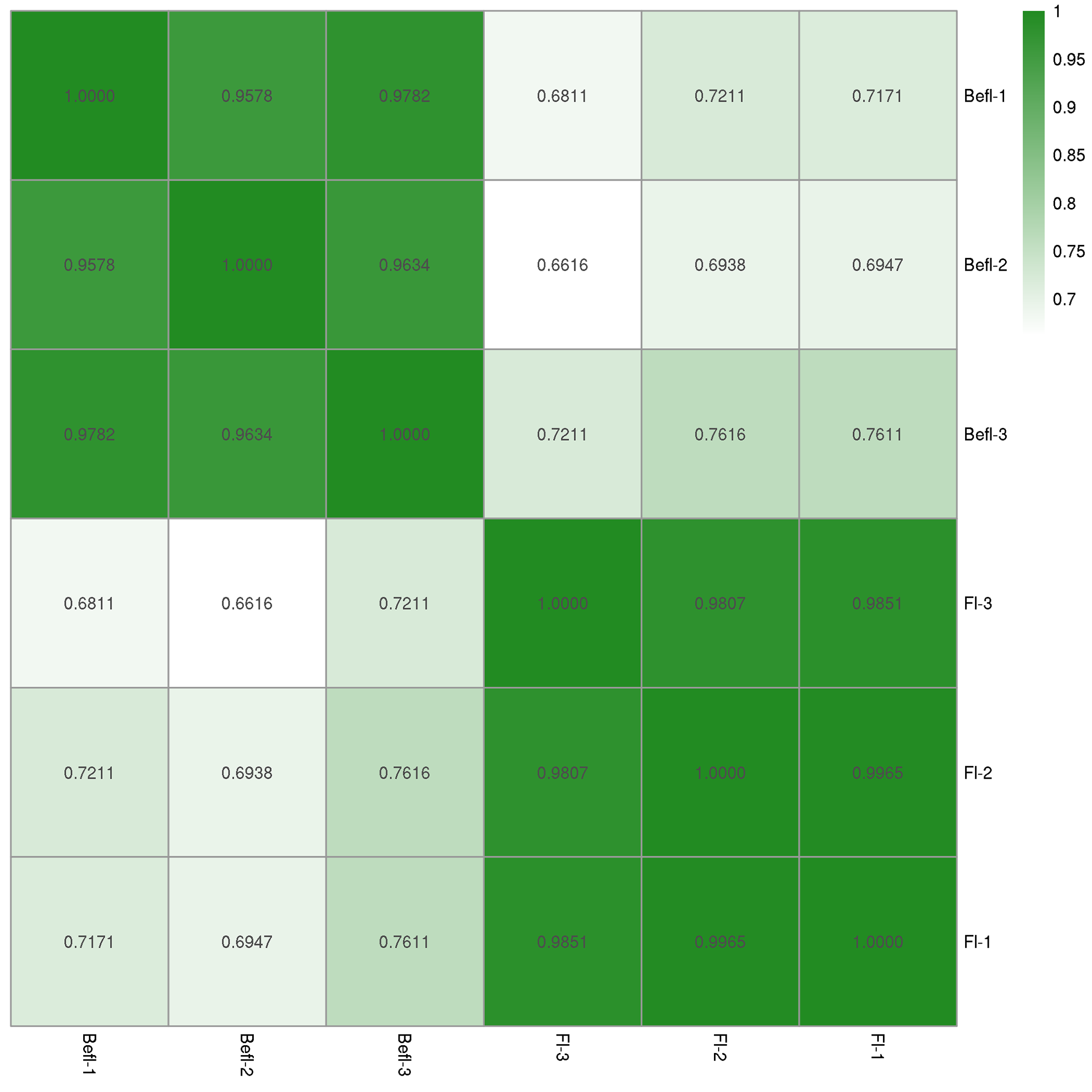


**Supplementary Figure S4** Pearson correlation heat map of all samples.


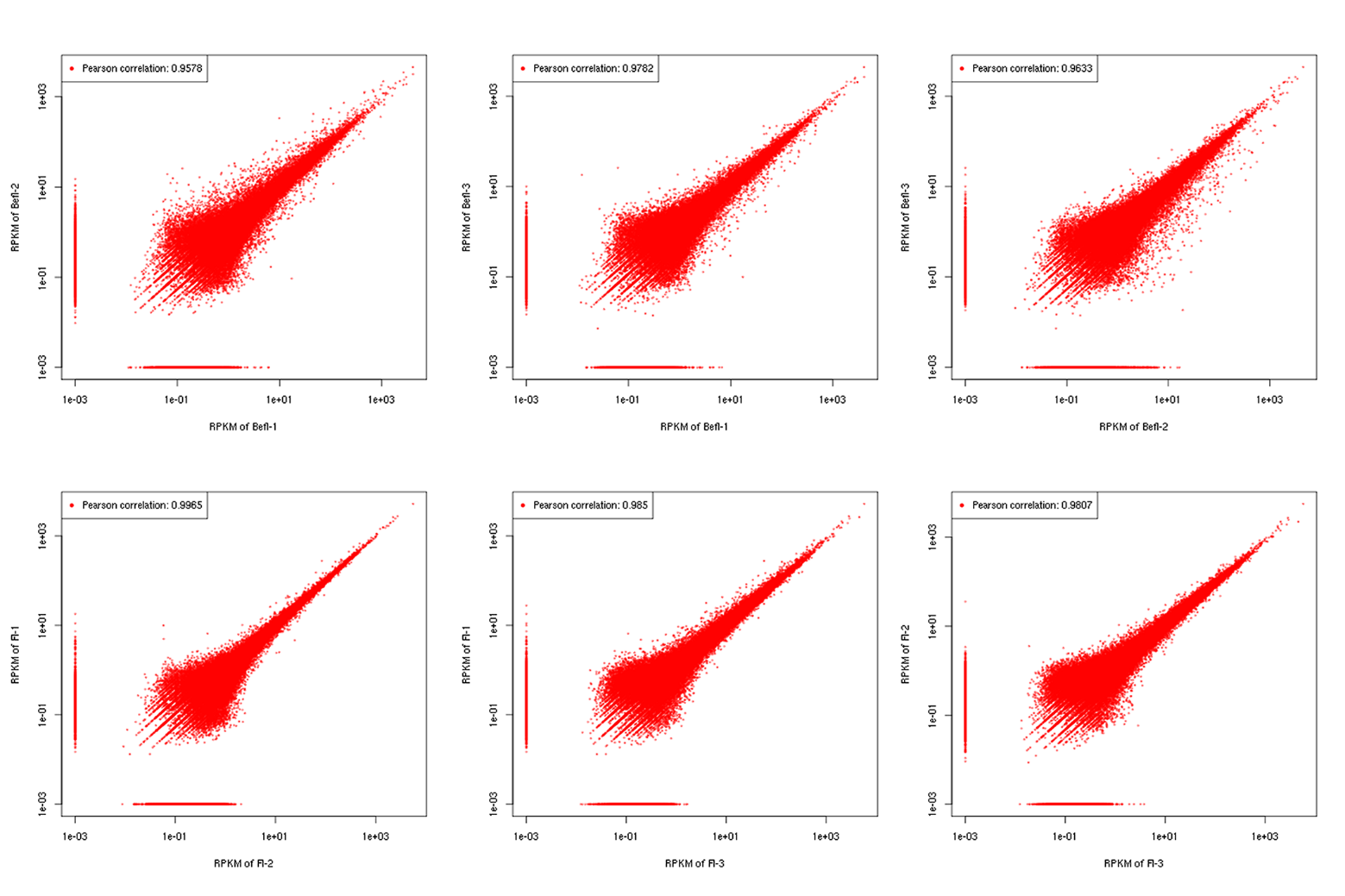


**Supplementary Figure S5** Pearson correlation for the parallel libraries.


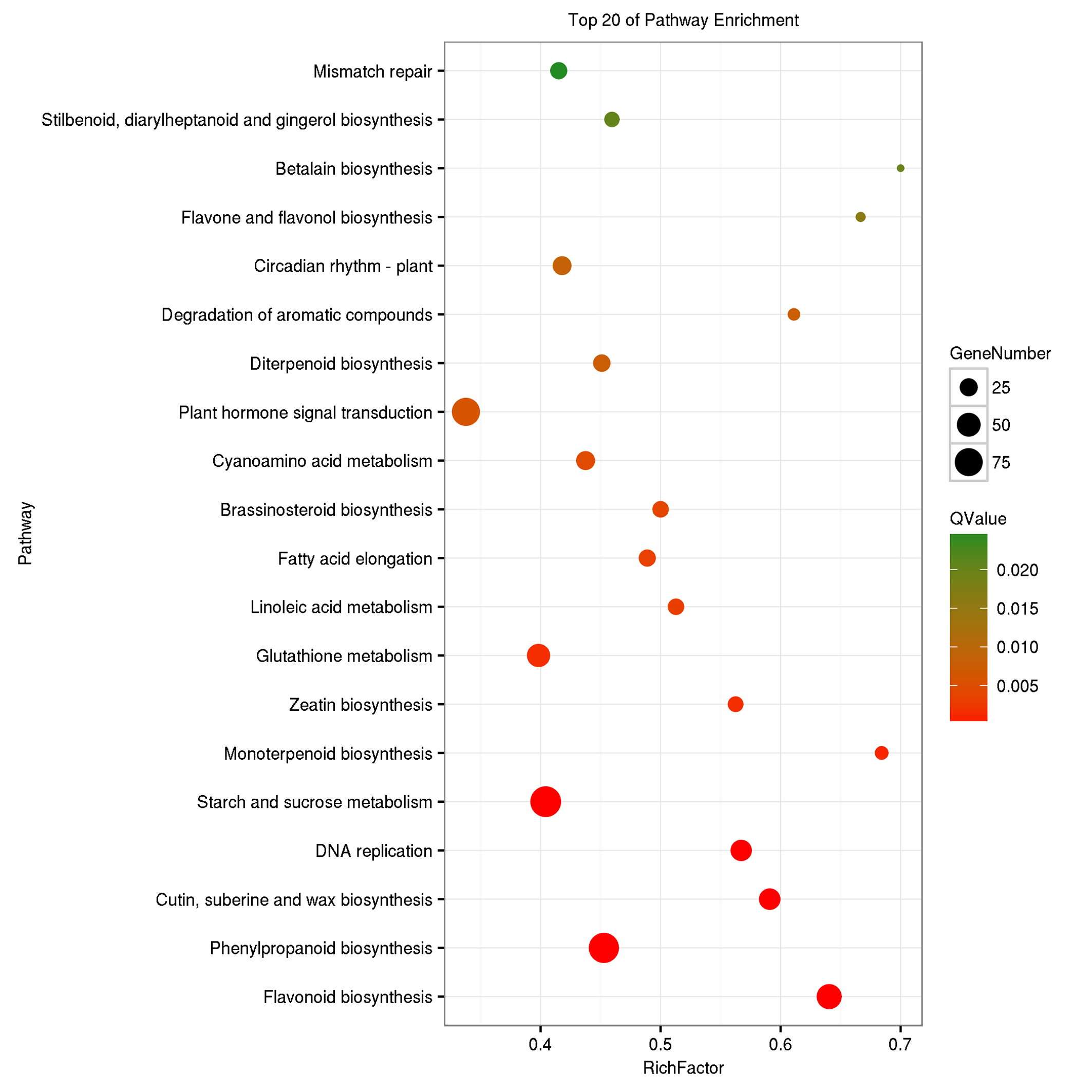


**Supplementary Figure S6** Top 20 of pathway enrichment.

**Supplementary Tables**

| **Supplementary Table S10 Changes in gene expression levels confirmed by qRT-PCR.** | | | | | | | |
| --- | --- | --- | --- | --- | --- | --- | --- |
| **Gene symbol** | **qPCR value** | **RNA-Seq value log_2_FC (Fl/Befl)** | **Size (bp)** | **Primer sequence (5’-3’)** |  | |  |
|  | **mean**±**SD** | **(mean)** |  |  |  | |  |
| *SWEET* | 0.372±0.233 | 1.1111531 | 108 | CACAGGACGGGCATTGTTTG | | Plus | |
|  |  |  |  | TCCACGCTTCTTGTCTCCAC | | Minus | |
| *DHN* | -5.611±1.195 | -7.617709 | 127 | TCTGCCCAGGAATCTTCTCC | | Plus | |
|  |  |  |  | CCAGATCAGCAGGAACGTGG | | Minus | |
| *CSLD* | 7.96±0.555 | 9.6218288 | 119 | TCAGGAGACGATCGGAAGGT | | Plus | |
|  |  |  |  | TGCCATCCAGGTAGCCTTTG | | Minus | |
| *CHS* | 2.904±0.577 | 3.4070097 | 169 | ACGCGTACTCGCCACTAAAG | | Plus | |
|  |  |  |  | GTGTTCGCTGTTGGTGATGC | | Minus | |
| *CHI* | 4.937±0.34 | 4.8773701 | 247 | TTGCTGGATTCGGAGTGACC | | Plus | |
|  |  |  |  | GCCTTGGAGAGTGAGAGCAG | | Minus | |
| *XYL* | 3.117±0.98 | 3.2717229 | 188 | GCAACGTGGCCAGTGTTATG | | Plus | |
|  |  |  |  | AACTGCATCTTCCGGAGTGG | | Minus | |
| *TSO* | 2.062±0.557 | 2.4468085 | 244 | ACTCACTCCAGCACTTCAGC | | Plus | |
|  |  |  |  | TGCCTGTGCACTTACAGGAG | | Minus | |
| *TLP* | 1.575±0.194 | 1.5585535 | 103 | ACTGTGTGGGTGGTGAAGTG | | Plus | |
|  |  |  |  | ATCCTGCCTCACAGGAGACT | | Minus | |
| *POD* | 2.858±1.002 | 3.1188893 | 199 | GCTTTGCTCACTTGTCTGCC | | Plus | |
|  |  |  |  | TCGACAGAGCCTTTGGACAC | | Minus | |
| *PAO* | 4.925±1.033 | 4.3069425 | 121 | TGAAGGACTCCAACGCTGAC | | Plus | |
|  |  |  |  | AGCTCAACAAGGTGGTACGG | | Minus | |
| *AGAL* | 1.439±0.499 | 1.5818117 | 87 | TGTGGAGCAGCAGAACAACT | | Plus | |
|  |  |  |  | TTCTTCAGTGACGGTCCAGG | | Minus | |
| *EXT* | -6.568±0.83 | -6.089029 | 217 | GGCCTGTCCAGTGGCTTTAT | | Plus | |
|  |  |  |  | CTACCACAGCCTTCCACAGG | | Minus | |
| *CRRSP* | 1.293±0.626 | 1.626144 | 124 | GATGAGGATGAGGCGCAACT | | Plus | |
|  |  |  |  | GGCATTGTTGCAGACAGACC | | Minus | |
| *GLIP* | 5.457±0.891 | 5.5319334 | 210 | GGAAGACTGACGCGGATGAT | | Plus | |
|  |  |  |  | TCCCATGGTGTAAAGTCGCC | | Minus | |
| *AP* | 1.173±0.181 | 1.370755 | 220 | TCAGCTCGTAGTCCTCGACA | | Plus | |
|  |  |  |  | GTACGATCCGTCGCCGTAAT | | Minus | |

**Supplementary Table S11 Protein in pollination drop.**

**A** Results of the identification of MS/MS.

| Sample | Total spectra | Identified spectra | Identified peptides | Identified proteins | Query number | Peptide seq | PSM FDR | Ions score | Protein ID | Modification |
| --- | --- | --- | --- | --- | --- | --- | --- | --- | --- | --- |
| PD1 | 3056 | 2 | 1 | 1 | 849 | EGNEVIAEATK | 0.016902074 | 21.7 | gi|7240303|gb|AAA84343.2| | - |
|  |  |  |  |  | 851 | EGNEVIAEATK | 0.036219296 | 18.39 | gi\|7240303\|gb\|AAA84343.2\| | - |
| PD2 | 7056 | 2 | 2 | 2 | 2185 | EGNEVIAEATK | 0.016441445 | 21.82 | gi|7240303|gb|AAA84343.2| | - |
|  |  |  |  |  | 4172 | QSNPNEQNVELNR | 0.030716236 | 16.74 | gi\|138277508\|gb\|AAQ09351.2\| | Gln->pyro-Glu (N-term Q)@N term |
| PD3 | 6943 | 1 | 1 | 1 | 1839 | IGSILNLYR | 0.016871355 | 22.78 | gi|512404594|gb|AGO02736.1| | Deamidated (NQ)@6N |

**B** List of protein identification.

| Group | Protein ID | Protein Qscore | Protein Mass | Peptide Seq | Coverage | Peptide Query | Description |
| --- | --- | --- | --- | --- | --- | --- | --- |
| 1 | gi|7240303|gb|AAA84343.2| | 1.742667306 | 52065.13909 | EGNEVIAEATK | 0.0213 | 849; 851; 2185 | ribulose 1,5-bisphosphate carboxylase, partial (chloroplast) [*Libocedrus plumosa*] |
| 2 | gi|138277508|gb|AAQ09351.2| | 1.489986373 | 4487.33511 | QSNPNEQNVELNR | 0.3421 | 4172 | photosystem II subunit L (chloroplast) [*Widdringtonia cedarbergensis*] |
| 3 | gi|512404594|gb|AGO02736.1| | 1.731457336 | 69465.98564 | IGSILNLYR | 0.0149 | 1839 | sabinene synthase [*Thuja plicata*] |

**Table notes**:

1. **Pollination drop material collection**

The pollination drops of the ovular tips of thousands of *P. orientalis* grown in the park were continuously collected every morning using glass capillary tubes that were ﬂame-drawn to a ﬁne point for precision collection and were concentrated into 1 mL plastic vials (3 replicates) from March 16 to 30, 2016. The vials were then wrapped in tin foil and stored in liquid nitrogen until analysis.

1. **Protein identification methods of pollination drop**

Liquid samples were supplemented with 25 mM NH4HCO3 with 10 mM DTT (final concentration) at 56 °C for 1 h, followed by the addition of 25 mM NH4HCO3 with 55 mM IAM (final concentration) and incubation in the dark for 45 min. After digestion with trypsin (1:40) at 37 °C for 8 h, the samples were desalted and vacuum-dried.

The peptide samples were reconstituted with mobile phase A (2% ACN, 0.1% FA) and centrifuged at 20,000 g for 10 min. The supernatants were collected for separation using a nanolitre liquid chromatograph (LC-20AD, Shimadzu, JP). After enrichment and desalting in a trap column, the samples were separated in series with a self-loaded C18 column (75 micron internal diameter, 3.6 micron size, 15 cm length) at 300 nl/min by the effective gradient: 0-8 min, 5% mobile phase B (98% CAN, 0.1% FA); 8-43 min, mobile phase B from 8% to 35% linearly; 43-48 min, mobile phase B from 35% to 60%; 48-50 min, mobile phase B from 60% to 80%; 50-55 min, 80% mobile phase B; 55-65 min, 5% mobile phase B.

Ionized by a nanoESI source, the separated peptides were detected in data-dependent acquisition (DDA) mode in a tandem mass spectrometer (MS/MS) (Q-Exactive, Thermo Fisher Scientific, San Jose, CA) with set parameters (1.6 kV ion source voltage, 350~1600 m/z MS scan range, 70,000 resolution, 100 secondary MS starting m/z; 17,500 resolution, charge 2+ to 7+ and the top 20 of peak intensity exceeding 10,000 of parent ion screening conditions for the secondary fragmentation, 27 HCD Collision Energy (NCE) and fragment ions in Orbitrap, 15 s dynamic exclusion time, level 1 3E6 and level 2 1E5 of AGC).

Based on databases such as UniProt, NCBI and Ensembl, the MS data were searched using Mascot 2.3.02. Due to the small amount of data, the results were filtered using a Mascot evalue<=0.05. The peptides were then inferred based on the parsimony principle to produce a series of proteomes.
